# Multilocus sequence typing of *Candida albicans* isolates from the oral cavities of patients undergoing haemodialysis

**DOI:** 10.1038/s41598-018-34565-7

**Published:** 2018-11-06

**Authors:** Yan-Bing Gong, Bo Jin, He Qi, Rong Zhang, Xiu-Ying Zhang, Ping Yuan, Tong-Xiang Zhao, Xing-Hua Geng, Min Zhang, Jian-Ling Zheng

**Affiliations:** 1 Department of Science Research, Peking University Shougang Hospital, Beijing, 100144 China; 2 Department of Clinical Laboratory, Peking University First Hospital, Beijing, 100034 China; 3 Department of Medical Biotechnology, Medical Sciences Institute of Liaoning, Shenyang, Liaoning Province 110101 China; 4 Department of Oral Medicine, Peking University Shougang Hospital, Beijing, 100144 China; 5 Department of Medical Record, Peking University Shougang Hospital, Beijing, 100144 China; 6 Department of Nephrology, Peking University Shougang Hospital, Beijing, 100144 China; 7 Department of Nephrology, Rehabilitation Hospital of Capital Medical University, Beijing, 100044 China

## Abstract

This study evaluates the prevalence, diversity, and genetic profiles of *Candida albicans* isolates recovered from the oral cavities of haemodialysis patients. Oral swab samples were obtained from haemodialysis patients (n = 126) and healthy control subjects (n = 233) and *Candida* species were characterised. There was no significant difference between the haemodialysis and control groups in the prevalence of yeast carriers (23.6% *vs*. 31.0%, respectively) or *C. albicans* carriers (19.8% *vs*. 21.0%, respectively). *C. albicans* was the most populous species in both cohorts, followed by *C. parapsilosis*. *C. parapsilosis* and *C. glabrata* were more prevalent in the haemodialysis group than in the control group (*C. parapsilosis* 5.6% *vs*. 0.9% and *C. glabrata* 3.2% *vs*. 0.4%, respectively; *P* < 0.05). *C. albicans* isolates were analysed by multilocus sequence typing and the results were used to construct a phylogenetic tree. Most haemodialysis isolates were placed into Clade 4 (20.0%) and Clade 19 (16.0%) and most control isolates into Clade 8 (17%) and Clade 4 (14.9%). Differences in the strain abundance in each clade were not statistically significant between the two groups. Moreover, there was no significant association between the health status or diagnosis and either the sequence types or clades.

## Introduction

In recent years, chronic kidney disease (CKD) has increased in both incidence and prevalence, and has become a worldwide public health problem. However, the lives of more than one million people worldwide have been extended by renal replacement therapy (haemodialysis or renal transplantation)^1–6^. A large-scale national survey in China in 2012 reported a prevalence of CKD of 10.8%^7,8^. CKD patients display an increased susceptibility to infection that may be related to uraemia-associated immunosuppression, similar to that observed in patients rendered immunodeficient following treatment with immunosuppressive agents. Moreover, chronic renal failure predisposes patients to opportunistic infections, mainly of fungal origin^9^, and haemodialysis has been identified as a major risk factor for candidaemia^10,11^. Most of these opportunistic infections are cutaneous and affect the moist mucosal membranes, especially those of the oral cavity^12–14^.

*Candida* species are commensal microbiota found in the oral cavities of 30–100% of humans^15–19^. *C. albicans* is widely recognised as the most pathogenic yeast species and is most commonly implicated in superficial and systemic infections^20–25^. Other *Candida* species, such as *C. glabrata*, *C. parapsilosis*, and *C. tropicalis*, are also associated with most forms of candidiasis^26–28^. However, the relative abundance of *Candida* species in the oral cavity is influenced by geographic location, health status and diagnosis, and previous exposure to antifungal drugs^10,29–31^.

In contrast to bacteria^32^, studies of yeast colonisation of the oral cavity of haemodialysis patients are rare. However, the previous studies all reported that *C. albicans* is more prevalent among the oral flora of haemodialysis patients than of healthy controls^2,33–35^. To address this knowledge gap, the objective of this study was to assess and compare the prevalence of yeast colonisation in the oral cavities of haemodialysis patients and healthy individuals. We report here the identities of the yeast species, their relative abundance, and their genetic profiles.

## Results

### Prevalence of yeast in oral swabs from the haemodialysis patient group

We recruited 126 haemodialysis patients at the Peking University Shougang Hospital in Beijing City, China, and obtained oral swabs from all patients within an 8-day period. Of the 126 patients examined, 39 (31.0%) were colonised by yeast and the majority of those (92.3%, 36/39) were colonised by a single species. The most prevalent species was *C. albicans* (64.1%, 25/39), which was present alone in 23 subjects and in combination with *C. glabrata* or *C. inconspicua* in 2 subjects. *C. parapsilosis* was identified in 17.9% (7/39) of carriers, *C. glabrata* in 10.3% (4/39), *C. tropicalis* in 5.1% (2/39), *Clavispora lusitaniae* in 5.1% (2/39), *C. inconspicua* in 2.6% (1/39), and *Meyerozyma guilliermondii* in 2.6% (1/39).

### Prevalence of yeast in oral swab samples from the control group

The control group comprised 233 subjects attending dental clinics for the treatment of dental caries at Peking University Shougang Hospital. Oral swabs were obtained over a 10-month period. Fifty-five (23.6%) subjects were colonised by yeast species. Similar to the patient group, *C. albicans* was the most prevalent yeast species (89.1%, 49/55) and was present alone in 46 subjects or in combination with *C. parapsilosis*, *C. tropicalis*, or *Clavispora lusitaniae* in 3 subjects. *C. parapsilosis* was identified in 3.6% (2/55) of carriers, *C. inconspicua* in 3.6% (2/55), *C. tropicalis* in 1.8% (1/55), *C. glabrata* in 1.8% (1/55), *Saccharomyces cerevisiae* in 1.8% (1/55), and *Yarrowia lipolytica* in 1.8% (1/55).

### Differences in yeast species between the haemodialysis patients and control subjects

**Figure 1:**
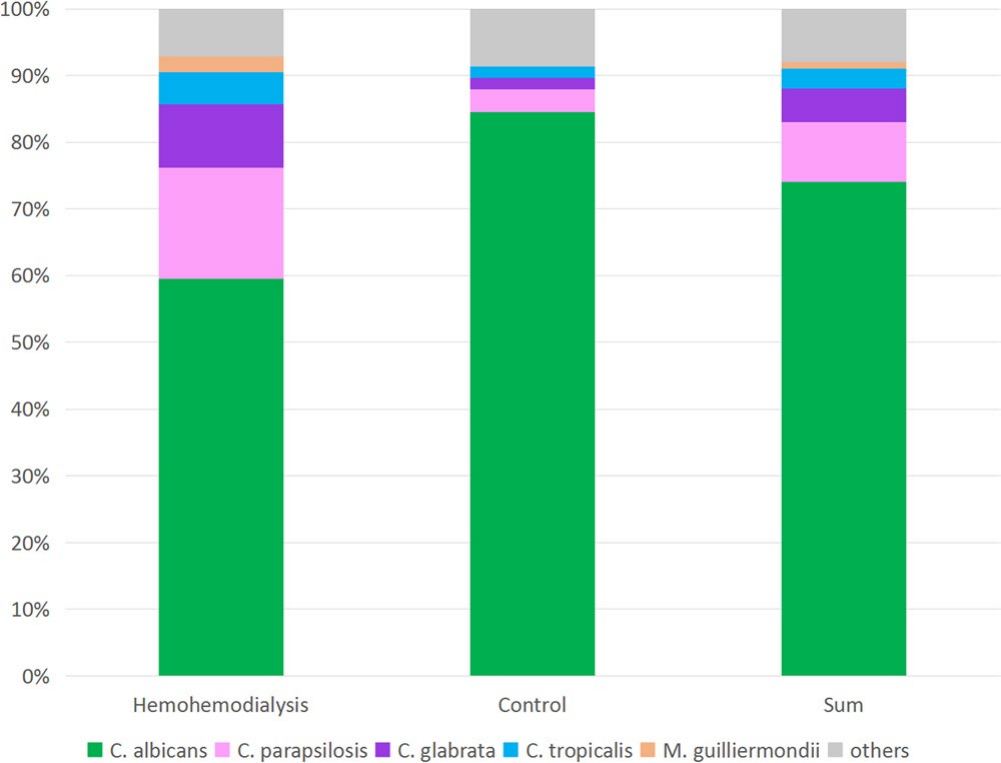
Composition of yeast species in the oral cavities of haemodialysis patients and control subjects. *C. albicans* and *C. parapsilosis* were the two most common yeast species in both groups. Yeast carriers in the haemodialysis and control groups differed significantly in the percentage carrying *C. albicans* (64.1% *vs*. 89.1%, *χ*^2^ = 8.507, *P* = 0.004) and *C. parapsilosis* (17.9% *vs*. 3.6%, *χ*^2^ = 5.399, *P* = 0.020). Five subjects were colonised by *C. albicans* in combination with other species: *C. glabrata* or *C. inconspicua* by two subjects in the haemodialysis group, and *C. parapsilosis*, *C. tropicalis*, or *Clavispora lusitaniae* by three subjects in the control group.

There were no significant differences between the percentage of haemodialysis patients and control subjects who were yeast carriers (31.0% *vs*. 23.6%, respectively) or *C. albicans* carriers (19.8% *vs*. 21.0%, respectively). However, *C. parapsilosis* and *C. glabrata* were both significantly more prevalent in the haemodialysis group compared with the control group (*C. parapsilosis*, 5.6% *vs*. 0.9%, *χ*^2^ = 7.382, *P* = 0.007; and *C. glabrata* 3.2% *vs*. 0.4%, *χ*^2^ = 4.488, *P* = 0.034). As shown in Fig. 1, the relative abundance of *C. albicans* and *C. parapsilosis* was also significantly difference in yeast carriers in the haemodialysis group compared with the control group (*C. albicans* 64.1% *vs*. 89.1%, *χ*^2^ = 8.507, *P* = 0.004; and *C. parapsilosis* 17.9% *vs*. 3.6%, *χ*^2^ = 5.399, *P* = 0.020, respectively). Kendall’s bivariate correlation analysis found no significant differences in the composition of yeast between the two groups and gender, age, diagnosis (glomerulonephritis, hypertension, coronary heart disease, diabetes, renal anaemia, secondary hyperparathyroidism), or other aspects of health status.

### Multilocus sequence typing (MLST) of *C. albicans* isolates

To perform MLST, *C. albicans* isolates from the patient and control groups were grown in culture dishes, and a single clone per dish was randomly chosen for MLST (total 25 and 49 clones from the haemodialysis and control groups, respectively). Two of the 74 samples (D1 and D132 from the control group) failed to yield PCR products from all seven loci; thus, MLST was performed on a total of 72 samples (25 from haemodialysis patients and 47 from controls). The analysis yielded 64 distinct sequence types (STs). Of these, 36 STs have not previously been identified (ST2837–ST2846 from the haemodialysis group; ST2611–ST2635 and ST2847 from the control group).

As shown in Supplementary Table S1 online, 57 of the 64 STs were identified from single isolates, and 7 were shared by multiple isolates (ST124 in D48a and N27a; ST299 in N12c and N23a; ST344 in N25b and N99a; ST768 in D16a and D30a; ST1608 in D93b and D114b); ST1865 in D23a, D133b, and N83b; and ST2454 in D43a and N54a). It seems unlikely that the strains sharing STs were derived from nosocomial infections for two reasons. First, most strains were isolated from the control group on different days, and the possibility that the subjects were in contact was small. Second, although all strains from the haemodialysis group were isolated on the same day, most of them had different STs, with the exception of ST299 (shared by N12c and N23a isolates) and ST344 (N25b and N99a). In addition, the patients who shared ST299 (N12 and N23) and ST344 (N25 and N99) were not in adjacent beds, making cross-infection unlikely. If the strains had been transferred by hospital staff, it would be expected that more patients would carry the same strains and that more strains would be shared by patients in adjacent beds than in remote beds.

**Figure 2:**
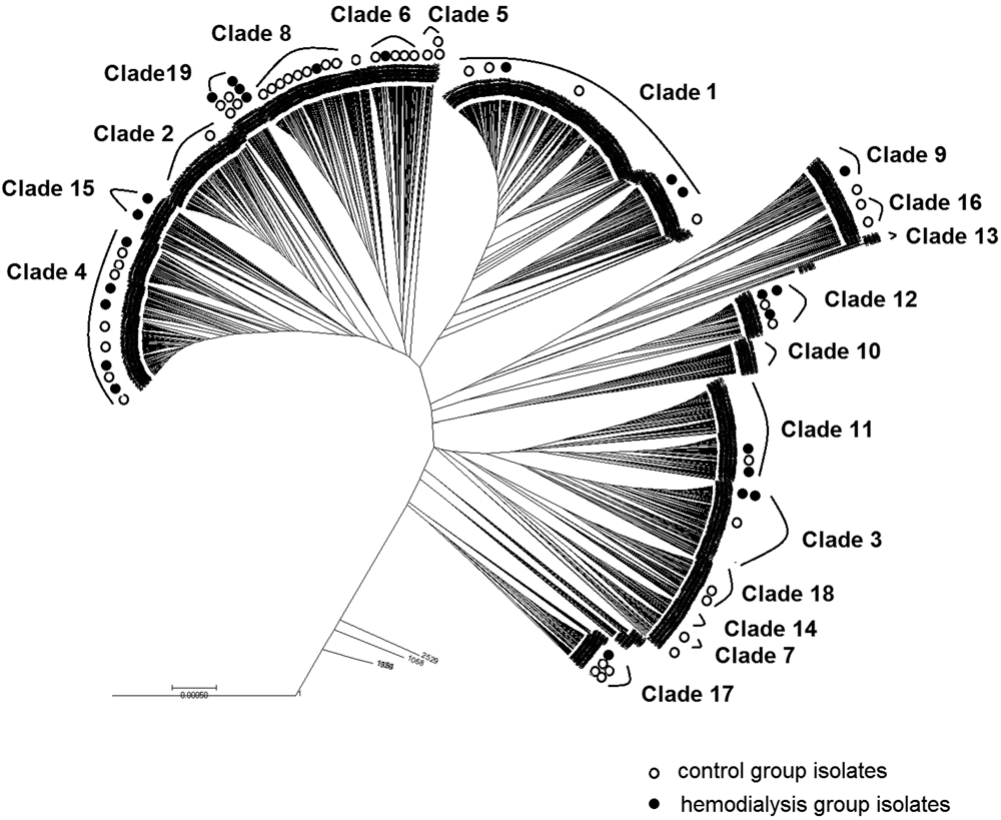
Radial distribution of *C. albicans* isolates from the haemodialysis and control groups and reference strains. Allelic concatenated nucleic acid sequences from 25 haemodialysis patient isolates, 47 control subject isolates, and 3256 reference strains retrieved from an MLST database were phylogenetically analysed by UPGMA. Clade numbers were assigned according to reference^41^. Three clades (10, 13, and 14) were not represented in isolates from either group. Open and closed circles represent control and haemodialysis isolates, respectively. Scale bar indicates p-distance.

To investigate the evolutionary relationships between the STs isolated in our study, we constructed an unweighted pair group method using average linkages (UPGMA) dendrogram of all *C. albicans* STs in the database (http://pubmlst.org/calbicans/). The dendrogram assigned the database STs to 19 clades plus singletons (Fig. 2 and Supplementary Fig. S1). Four of the STs identified here (ST2611, ST2634, ST2635, and ST2847) were singletons, and the other STs were distributed among 16 clades.

**Figure 3:**
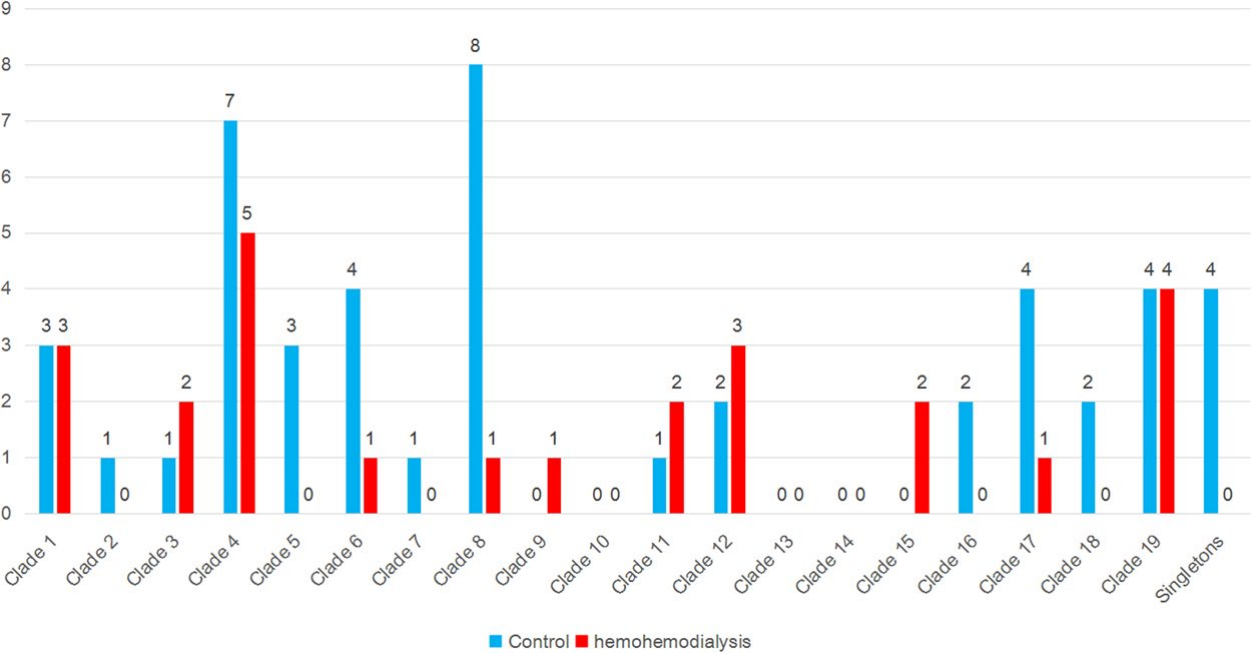
Number of *C. albicans* isolates in each clade isolated from the haemodialysis patients and control subjects. Red and blue bars indicate haemodialysis and control groups, respectively. There were no significant differences in clade abundance between the two groups.

As shown in Fig. 3, there were no Clade 10, 13, or 14 strains identified in the haemodialysis or control groups in this study. Five clades (2, 5, 7, 16, and 18) included strains from the control group but not the haemodialysis group, and conversely, Clades 9 and 15 included strains only from the haemodialysis group. Nine clades (1, 3, 4, 6, 8, 11, 12, 17, and 19) included strains from both groups.

The most abundant *C. albicans* strains from the haemodialysis group were in Clade 4 (20.0%) and Clade 19 (16.0%) and the most abundant from the control group were in Clade 8 (17.0%) and Clade 4 (14.9%). There were no significant differences in the strain abundance in each clade between the two groups. Moreover, there were no significant associations between the diagnosis in the haemodialysis patient group and either the STs or clades, which is most likely due to the limited number of samples included in the analysis.

## Discussion

This study evaluated the prevalence, diversity, and genetic profiles of *C. albicans* isolates recovered from the naturally colonised oral mucosa of 126 haemodialysis patients and 233 volunteers visiting dentist clinics. We found that the prevalence of yeast and *C. albicans* carriers was similar among the haemodialysis patients and control subjects (yeast 31.0% *vs*. 23.6% and *C. albicans* 19.8% *vs*. 21.0%, respectively). These results differ from those of previous reports. In a Brazilian study, Bastos *et al*.^2^ reported that *C. albicans* was more common in the oral cavities of patients undergoing haemodialysis and peritoneal dialysis (72.7%) compared with a control group (26.3%). Similarly, a study in Iran by Ahmadieh *et al*.^33^ found that *Candida* species were more common in haemodialysis patients (38.8%) than control subjects (18.0%). In a Turkish study, Gulcan *et al*.^35^ reported oral yeast colonisation of 40% of haemodialysis patients and only 18% of healthy subjects. These differences may be related to various factors, including oral care, health habits, and other characteristics that differed in the populations studied. Watson and Kroone^36^ reported that host factors are important in resisting oral colonisation by *Candida*, and they suggested that *C. albicans*-exposed subjects who remain non-carriers may be able to eliminate the yeast. In general, oral candidiasis is much more prevalent in immunocompromised individuals, such as haemodialysis patients, than in healthy subjects^9,11^.

In our study, *C. albicans* was the most abundant yeast species in the oral cavities of both haemodialysis and control groups, followed by *C. parapsilosis* and *C. glabrata*. The distribution of oral yeast species identified here was similar to those in other reports^11^. Godoy *et al*.^3^ reported a distribution of 63% *C. albicans*, 16% *C. glabrata*, and 1.2% *C. parapsilosis* among yeast in the oral cavities of patients with chronic renal failure undergoing haemodialysis in Brazil. De la Rosa-García1 *et al*.^9^ reported that *C. albicans*, *C. glabrata*, and *C. parapsilosis* comprised 74.6%, 22.0%, and 3.4%, respectively, of oral *Candida* species isolated from haemodialysis and peritoneal dialysis patients in a Mexican study. The influence of geographic location and the specific patient cohort on the relative distribution of oral *Candida* species merits further research^9,10,29,31,33^.

We characterised the genetic diversity and population structure of *C. albicans* isolated from the haemodialysis and control groups by MLST. To investigate the evolutionary relationships between the STs, we constructed a UPGMA-based dendrogram of all *C. albicans* in the MLST database. In our study, we detected no isolates in Clades 10, 13, or 14 in either the patient or control group. Similarly, these clades contain the fewest strains in the global *C. albicans* MLST database. No isolates in Clade 13 were found in studies from Chinese regions such as Beijing^37^, Chengdu^38^, Shanghai^39,40^, Shenyang^41^, and Taiwan^42–44^. Several earlier studies found strong evidence for a geographic influence in the low representation of some clades in certain regions of the world. It has been suggested that this may result from a selection process under the constraint of climate zones. A better explanation might be the presence of a specific or prevalent host factor that diminishes the abundance of certain strain types within a population^45^.

It is generally thought that individuals are colonised by a single *C. albicans* strain or by several very similar strains^46^. Strains may persist over time and the same strain type can be present at multiple body sites in the same patient^44,46–54^. Therefore, we restricted the MLST analysis to only one isolate per individual. Although the average age of the haemodialysis and control groups in our study was different (59.76 *vs*. 42.79 years, respectively), the oral flora and genotype in adults should be stable. However, we cannot formally conclude that the age difference did not affect the distribution of strain genotypes in our patients and healthy subjects.

In our study, we identified more STs and clades in the *C. albicans* isolates from the control group than the haemodialysis group, and the clade distribution was different in the two groups. Clade 4 was the most populous clade in the haemodialysis group, the second most populous in the control group, and the second most populous in the global database^43^. Conversely, Clade 8 was the most populous clade in the control group, the third most populous in the global database, but did not rank in the three most abundant clades in the haemodialysis group.

Clade 19 was the second most populous clade in the haemodialysis group but was less abundant in the control group. In the global database, most Clade 19 isolates were from Asian subjects (China, South Korea, Iran, Kuwait). This clade was first described as clade *New 1* by Wu^40^ in Shanghai, China.

Clades 9 and 15 included strains from the haemodialysis group but not the control group. We speculate that immunocompromised patients may be more vulnerable than their healthy counterparts to colonisation by Clade 15 *C. albicans* strains, because Clade 15 was also reported to be more populous in autoimmune polyendocrinopathy-candidiasis-ectodermal dystrophy patients than in healthy subjects in an Irish study^51^. Although the sample number in our study was insufficient to demonstrate statistical significance, our finding implies that an individual’s health status affects the clade distribution of *C. albicans* strains isolated from the oral cavity.

The clade and ST distributions in the *C. albicans* MLST database show clear effects of geography and ethnicity, but the differences are gradually being reduced by globalization^55^. Several studies of geographically close populations indicate that *C. albicans* STs concentrate within certain clades that reflect not only the geographical location but also the host’s physical status. For example, *C. albicans* isolates obtained from blood cultures from healthy individuals in Scotland^56^, the oral cavities of students from throughout the UK^57^, and the oral cavities of chronic mucocutaneous candidiasis patients throughout the UK^54^ were mainly grouped into Clades 1, 2, and 4 (not necessarily in that order of abundance). However, *C. albicans* isolates from intensive care unit patients in Leeds, England, were concentrated in Clades 2, 1, and 3^46^. Studies performed in Ireland have confirmed that clade distributions differ among patients with different disorders. In the Irish study, the top three clades (in no specific order) were 2, 1, and 4 in healthy subjects, 1, 11, and 4 in periodontitis patients^15^, 1, 3, and 8 in leukoplakia patients^20^, and 1, 4, and 15 in autoimmune polyendocrinopathy-candidiasis-ectodermal dystrophy patients^51^. Our study is thus consistent with the reports from the UK and Ireland and collectively showed that (i) Clade 1 or 4 was the most populous clade in both the control and patient groups, (ii) the second and third most populous clades differed between the patients and healthy subjects, and (iii) the most scarce clades were more common in the patient group than in the control group. These results suggest that strains within a particular geographical locale are genetically more similar, on average, than strains from distant locales, and additionally that the genetic similarity is more strongly affected by geographical location than by health status or disease^58^.

There were some limitations to this study. First, it was a single-centre study and the results may be less generalisable than those from multi-centre studies^42^. Second, not all isolates from this study were available for genotyping. Third, the relatively small numbers of patients and controls in this study provided insufficient statistical power to compare several parameters between the control and haemodialysis groups (e.g., *C. albicans* STs) or to evaluate the associations between molecular types and clinicopathological data (e.g., gender, age, diagnosis). Finally, although MLST is well known for its high discriminatory power and reproducibility, we did not use a second, complementary, method to MLST to verify identical STs shared by more than one isolate.

To our knowledge, this is the first MLST characterisation of *C. albicans* isolated from the oral cavities of patients on haemodialysis. Future studies implementing higher throughput genome-wide sequencing, together with more sophisticated models of the population dynamics of medically relevant yeast^54^, will improve our understanding of the population dynamics of fungal infections in diverse epidemiological settings and will inform our approach to preventative and therapeutic treatments and strategies. It would also be interesting to investigate the degree to which *C. albicans* genotypes influences infection susceptibility in haemodialysis patients from different ethnic or geographical populations.

## Methods

### Study participants and isolates

Written informed consent was obtained from all study participants or from the parents of minors prior to obtaining samples. This study was approved by the Peking University Shougang Hospital Institutional Review Board (protocol number 2013–85). For the patient group, oral swab samples were collected from 126 haemodialysis patients at the Peking University Shougang Hospital in Beijing City, China. The medical history of each patient was recorded. The mean age was 59.76 years (median 60 years, range 28–82 years) and the gender distribution was 75 (59.5%) male and 51 (40.5%) female. The samples were collected between May 15, 2014 and May 23, 2014. For the control group, oral swab samples were collected from 233 volunteers attending dental clinics for the treatment of dental caries at the Peking University Shougang Hospital in Beijing City, China. A comprehensive dental and medical history was recorded for each patient. The mean age was 42.79 years (median 41 years, range 17–85 years) and the gender distribution was 104 (44.6%) male and 129 (55.4%) female. The samples were collected between May 18, 2013 and February 28, 2014.

### Culture and identification of *Candida* species

Oral cavities were sampled with sterile cotton transport swabs^46,48,54,59^. Samples from each subject were plated on Sabouraud Dextrose Liquid Medium (Beijing Aoboxing Bio-Tech Co. Ltd., Beijing, China) and incubated at 37 °C for 24 h. Yeast colonies were then streaked onto CHROMagar medium (Beijing Aoboxing Bio-Tech Co. Ltd.) and incubated at 37 °C for 48 h^60^. After culturing for two days at 37 °C, we tentatively classified the yeast species by the colony colour as *C. albicans* (green), *C. tropicalis* (blue), *C. krusei* (pink), and *C. glabrata* (purple). However, since colour alone cannot accurately distinguish between some *C. albicans* and non-albicans species, we randomly picked one clone of each colour from the same dish for species identification by DNA analysis.

### DNA preparation

The yeast isolates were subcultured on Sabouraud Dextrose Liquid Medium (Beijing Aoboxing Bio-Tech Co. Ltd.) and incubated at 37 °C for 24 h. An aliquot (3 ml) of the cell suspension was microcentrifuged at 12,000 rpm for 30 s, and genomic DNA was extracted using a Yeast Genomic DNA Extraction Miniprep system (ABIgen Corp., Beijing, China) in accordance with the manufacturer’s instructions. DNA samples were stored at −20 °C until analysis.

### Amplification and sequencing of the internal transcribed spacer (ITS) regions

The fungus-specific universal primers ITS1 (5′-TCCGTAGGTGAACCTGCGG-3′) and ITS4 (5′-TCCTCCGCTTATTGATATGC-3′) were used to amplify the ITS1 and ITS2 regions^61–63^. PCR was performed using 2 × EasyTaq PCR SuperMix (Transgen Biotech Inc., Beijing, China) in accordance with the manufacturer’s instructions. The final PCR reaction volume was 50 μl and the conditions were: initial denaturation at 94 °C for 5 min; 30 cycles of denaturation (94 °C for 30 s), annealing (60 °C for 30 s), and extension (72 °C for 1 min); and a final extension step at 72 °C for 10 min. The DNA fragments were then sequenced (Tskingke Biological Technology Inc., Beijing, China). All amplicons were sequenced on both strands using ITS1 and ITS4 primers for the ITS1 and ITS2 regions. The ITS sequences of each type strain were submitted to GenBank to facilitate sequence comparison of strains belonging to these species.

### Identification of yeast by ITS sequencing

A total of 191 strains were examined. The species were identified using the BLAST sequence analysis tool (http://www.ncbi.nlm.nih.gov/BLAST/). The ITS sequence was compared using nucleotide-nucleotide BLAST (blastn) with default settings, except that the sequences were not filtered for low complexity. Species identification was determined from the lowest expected value of the BLAST output. Occasionally, the BLAST search returned sequences from two different species with 100% identity. In these cases, the ITS1 and ITS2 lengths were taken into consideration, since they are important characteristics of fungal species^64^. The GenBank accession numbers of the ITS1 and ITS2 regions of type strains of 191 species sequenced in this study are given in Supplementary Table S1.

### MLST and data analysis

*C. albicans* samples isolated from 25 haemodialysis patients and 49 control subjects (74 samples in total) were grown in dishes, and one clone from each dish was randomly chosen for MLST as described previously^65–67^. MLST was performed by amplifying seven *C. albicans* housekeeping genes (Ca AAT1a, Ca ACC1, Ca ADP1, Ca MPIb, Ca SYA1, Ca VPS13, and Ca ZWF1b) using the following specific primers: AAT1a Fwd 5′-ACTCAAGCTAGATTTTTGGC-3′ and Rev 5′-CAGCAACATGATTAGCCC-3′; ACC1 Fwd 5′-GCAAGAGAAATTTTAATTCAATG-3′ and Rev 5′-TTCATCAACATCATCCAAGTG-3′; ADP1 Fwd 5′-GAGCCAAGTATGAATGATTTG-3′ and Rev 5′-TTGATCAACAAACCCGATAAT-3′; MPIb Fwd 5′-ACCAGAAATGGCCATTGC-3′ and Rev 5′-GCAGCCATGCATTCAATTAT-3′; SYA1 Fwd 5′-AGAAGAATTGTTGCTGTTACTG-3′ and Rev 5′-GTTACCTTTACCACCAGCTTT-3′; VPS13 Fwd 5′-TCGTTGAGAGATATTCGACTT-3′ and Rev 5′-ACGGATGGATCTCCAGTCC-3′; and ZWF1b Fwd 5′-GTTTCATTTGATCCTGAAGC-3′ and Rev 5′-GCCATTGATAAGTACCTGGAT-3′.

PCR was performed using 2 × EasyTaq PCR SuperMix (Transgen Biotech Inc.) in accordance with the manufacturer’s instructions. The final PCR reaction volume was 50 μl and the conditions were: initial denaturation at 94 °C for 5 min; 30 cycles of denaturation (94 °C for 30 s), annealing (60 °C for 30 s), and extension (72 °C for 1 min); and a final extension step at 72 °C for 10 min. The DNA fragments were sequenced (Tskingke Biological Technology Inc.) using the same primers as for PCR, and all seven loci were sequenced in both directions. Samples D1 and D132 from the control group failed to yield PCR products for all seven loci; thus, complete MLST was achieved on a total of 72 samples (25 haemodialysis patients and 47 controls).

For each gene, distinct alleles were identified and numbered using the nonredundant database program at the MLST website (http://pubmlst.org/calbicans/). The alleles at each of the seven loci constituted a strain’s allelic profile (i.e., the ST), and each distinct allelic profile was considered a unique ST or genotype. A dendrogram based on the pairwise differences in the allelic profiles of the seven genes was constructed by UPGMA analysis using MLSTest software (http://www.ipe.unsa.edu.ar/software), which compares genotypic profiles of global isolates^68–70^.

### Statistical analysis

Differences were compared using Pearson’s chi-squared test and associations were evaluated by Kendall’s bivariate correlation analysis. SPSS version 19 software (IBM Corp., Armonk, NY, USA) was used for all analyses. The results were considered statistically significant at the *P* < 0.05 level.

## Electronic supplementary material

Table S1

Figur S1

## References

[1] Arora P (2013). Prevalence estimates of chronic kidney disease in Canada: results of a nationally representative survey. CMAJ..

[2] Bastos JA (2011). Identification of periodontal pathogens and severity of periodontitis in patients with and without chronic kidney disease. Arch. Oral Biol..

[3] Godoy JS (2013). Colonization of the oral cavity by yeasts in patients with chronic renal failure undergoing hemodialysis. J. Oral Pathol. Med..

[4] Hallan SI (2006). International comparison of the relationship of chronic kidney disease prevalence and ESRD risk. J. Am. Soc. Nephrol..

[5] Hoerger TJ (2015). The future burden of CKD in the United States: a simulation model for the CDC CKD Initiative. Am. J. Kidney Dis..

[6] Perico N (2005). Strategies for national health care systems in emerging countries: the case of screening and prevention of renal disease progression in Bolivia. Kidney Int. Suppl..

[7] Kong X (2013). Association between family members of dialysis patients and chronic kidney disease: a multicenter study in China. BMC Nephrol..

[8] Zhang L (2012). Prevalence of chronic kidney disease in China: a cross-sectional survey. Lancet..

[9] de la Rosa-Garcia E, Miramontes-Zapata M, Sanchez-Vargas LO, Mondragon-Padilla A (2013). Oral colonisation and infection by Candida sp. in diabetic and non-diabetic patients with chronic kidney disease on dialysis. Nefrologia..

[10] Conde-Rosa A (2010). Candidemia distribution, associated risk factors, and attributed mortality at a university-based medical center. P. R. Health Sci. J..

[11] Pieralisi N, Godoy J, Yamada S, Santana R, Svidzinski T (2015). Oral lesions and colonization by yeasts in hemodialysis patients. J. Oral Pathol. Med..

[12] Williams DW, Kuriyama T, Silva S, Malic S, Lewis MA (2011). Candida biofilms and oral candidosis: treatment and prevention. Periodontol. 2000..

[13] Fidel PJ (2006). Candida-host interactions in HIV disease: relationships in oropharyngeal candidiasis. Adv. Dent. Res..

[14] Olczak-Kowalczyk D (2012). Bacteria and Candida yeasts in inflammations of the oral mucosa in children with secondary immunodeficiency. J. Oral Pathol. Med..

[15] McManus BA (2012). Enrichment of multilocus sequence typing clade 1 with oral Candida albicans isolates in patients with untreated periodontitis. J. Clin. Microbiol..

[16] Samaranayake L (2009). Commensal oral Candida in Asian cohorts. Int. J. Oral Sci..

[17] Dongari-Bagtzoglou A, Fidel PJ (2005). The host cytokine responses and protective immunity in oropharyngeal candidiasis. J. Dent. Res..

[18] Qi QG, Hu T, Zhou XD (2005). Frequency, species and molecular characterization of oral Candida in hosts of different age in China. J. Oral Pathol. Med..

[19] Imabayashi Y (2016). Molecular analysis of fungal populations in patients with oral candidiasis using next-generation sequencing. Sci. Rep..

[20] Abdulrahim MH, McManus BA, Flint SR, Coleman DC (2013). Genotyping Candida albicans from Candida leukoplakia and non-Candida leukoplakia shows no enrichment of multilocus sequence typing clades but enrichment of ABC genotype C in Candida leukoplakia. Plos One..

[21] Fidel PJ (2011). Candida-host interactions in HIV disease: implications for oropharyngeal candidiasis. Adv. Dent. Res..

[22] Gainza-Cirauqui ML (2013). Production of carcinogenic acetaldehyde by Candida albicans from patients with potentially malignant oral mucosal disorders. J. Oral Pathol. Med..

[23] Luan C (2015). Dysbiosis of fungal microbiota in the intestinal mucosa of patients with colorectal adenomas. Sci. Rep..

[24] Lopes JP, Stylianou M, Nilsson G, Urban CF (2015). Opportunistic pathogen Candida albicans elicits a temporal response in primary human mast cells. Sci. Rep..

[25] Wilson MJ, Williams DW, Forbes MD, Finlay IG, Lewis MA (2001). A molecular epidemiological study of sequential oral isolates of Candida albicans from terminally ill patients. J. Oral Pathol. Med..

[26] Salvatori O, Puri S, Tati S, Edgerton M (2016). Innate Immunity and Saliva in Candida albicans-mediated Oral Diseases. J. Dent. Res..

[27] Ten CJ, Klis FM, Pereira-Cenci T, Crielaard W, de Groot PW (2009). Molecular and cellular mechanisms that lead to Candida biofilm formation. J. Dent. Res..

[28] Wu JY (2017). Multilocus sequence analyses reveal extensive diversity and multiple origins of fluconazole resistance in Candida tropicalis from tropical China. Sci. Rep..

[29] Pfaller MA, Diekema DJ (2007). Epidemiology of invasive candidiasis: a persistent public health problem. Clin. Microbiol Rev..

[30] Kullaa-Mikkonen A, Kotilainen R (1983). The prevalence of oral carriers of Candida in patients with tongue abnormalities. J. Dent..

[31] Pires RH, Santos JM, Zaia JE, Martins CH, Mendes-Giannini MJ (2011). Candida parapsilosis complex water isolates from a hemodialysis unit: biofilm production and *in vitro* evaluation of the use of clinical antifungals. Mem. Inst. Oswaldo Cruz..

[32] Shariff G (2004). Relationship between oral bacteria and hemodialysis access infection. Oral Surg. Oral Med. Oral Pathol. Oral Radiol. Endod..

[33] Ahmadieh A, Baharvand M, Fallah F, Djaladat H, Eslani M (2010). Oral microflora in patients on hemodialysis and kidney transplant recipients. Iran J. Kidney Dis..

[34] Takeuchi Y (2007). Study of the oral microbial flora in patients with renal disease. Nephrology (Carlton)..

[35] Gulcan A, Gulcan E, Keles M, Aktas E (2016). Oral yeast colonization in peritoneal dialysis and hemodialysis patients and renal transplant recipients. Comp. Immunol. Microbiol. Infect. Dis..

[36] Watson CJ, Kroone HB (1981). The survival of Candida albicans experimentally inoculated into the mouths of healthy human subjects. J. Dent..

[37] Ge S (2012). Prevalence of specific and phylogenetically closely related genotypes in the population of Candida albicans associated with genital candidiasis in China. Fungal Genet. Biol..

[38] Xiao YL (2012). Multilocus sequence typing of Candida albicans bloodstream isolates in an intensive care unit. Sichuan Da Xue Xue Bao Yi Xue Ban..

[39] Hu L (2015). Genetic and phenotypic characterization of Candida albicans strains isolated from infectious disease patients in Shanghai. J. Med. Microbiol..

[40] Wu K (2015). Multilocus Sequence Typing of Pathogenic Candida albicans Isolates Collected from a Teaching Hospital in Shanghai, China: A Molecular Epidemiology Study. Plos One..

[41] Gong YB (2012). Particular Candida albicans strains in the digestive tract of dyspeptic patients, identified by multilocus sequence typing. Plos One..

[42] Tsai MH (2015). Clinical and molecular characteristics of bloodstream infections caused by Candida albicans in children from 2003 to 2011. Clin. Microbiol. Infect..

[43] Wang SH (2015). Molecular epidemiology of invasive Candida albicans at a tertiary hospital in northern Taiwan from 2003 to 2011. Med. Mycol..

[44] Chen KW (2006). Multilocus sequence typing for analyses of clonality of Candida albicans strains in Taiwan. J. Clin. Microbiol..

[45] Da Matta D (2010). Candidemia surveillance in Brazil: evidence for a geographical boundary defining an area exhibiting an abatement of infections by Candida albicans group 2 strains. J. Clin. Microbiol..

[46] Cliff PR, Sandoe JA, Heritage J, Barton RC (2008). Use of multilocus sequence typing for the investigation of colonisation by Candida albicans in intensive care unit patients. J. Hosp. Infect..

[47] Afsarian MH, Badali H, Boekhout T, Shokohi T, Katiraee F (2015). Multilocus sequence typing of Candida albicans isolates from a burn intensive care unit in Iran. J. Med. Microbiol..

[48] Bougnoux ME (2006). Multilocus sequence typing reveals intrafamilial transmission and microevolutions of Candida albicans isolates from the human digestive tract. J. Clin. Microbiol..

[49] Choo, K. H., Lee, H. J., Knight, N. J., Holmes, A. R. & Cannon, R. D. Multilocus sequence typing (MLST) analysis ofCandida albicans isolates colonizing acrylic dentures before and after denture replacement. *Med. Mycol*. w128 (2016). 10.1093/mmy/myw128 27915298

[50] Chowdhary A (2006). Comparison of multilocus sequence typing and Ca3 fingerprinting for molecular subtyping epidemiologically-related clinical isolates of Candida albicans. Med. Mycol..

[51] McManus BA (2011). Microbiological screening of Irish patients with autoimmune polyendocrinopathy-candidiasis-ectodermal dystrophy reveals persistence of Candida albicans strains, gradual reduction in susceptibility to azoles, and incidences of clinical signs of oral candidiasis without culture evidence. J. Clin. Microbiol..

[52] Da Matta D (2010). Multilocus sequence typing of sequential Candida albicans isolates from patients with persistent or recurrent fungemia. Med. Mycol..

[53] Gammelsrud KW (2012). Multilocus sequence typing of serial Candida albicans isolates from children with cancer, children with cystic fibrosis and healthy controls. Med. Mycol..

[54] Moorhouse AJ, Rennison C, Raza M, Lilic D, Gow NA (2016). Clonal Strain Persistence of Candida albicans Isolates from Chronic Mucocutaneous Candidiasis Patients. Plos One..

[55] Takakura S (2008). Comparison of Candida albicans strain types among isolates from three countries. Int. J. Med. Microbiol..

[56] Odds FC (2007). One year prospective survey of Candida bloodstream infections in Scotland. J. Med. Microbiol..

[57] Jacobsen MD (2008). Mixed Candida albicans strain populations in colonized and infected mucosal tissues. FEMS Yeast Res..

[58] Schmid J, Rotman M, Reed B, Pierson CL, Soll DR (1993). Genetic similarity of Candida albicans strains from vaginitis patients and their partners. J. Clin. Microbiol..

[59] Wrobel L (2008). Molecular phylogenetic analysis of a geographically and temporally matched set of Candida albicans isolates from humans and nonmigratory wildlife in central Illinois. Eukaryot. Cell..

[60] Odds FC, Bernaerts R (1994). CHROMagar Candida, a new differential isolation medium for presumptive identification of clinically important Candida species. J. Clin. Microbiol..

[61] Chen YC (2001). Polymorphic internal transcribed spacer region 1 DNA sequences identify medically important yeasts. J. Clin. Microbiol..

[62] Schoch CL (2012). Nuclear ribosomal internal transcribed spacer (ITS) region as a universal DNA barcode marker for Fungi. Proc. Natl. Acad. Sci. USA.

[63] Zahir RA, Himratul-Aznita WH (2013). Distribution of Candida in the oral cavity and its differentiation based on the internally transcribed spacer (ITS) regions of rDNA. Yeast..

[64] Leaw SN (2006). Identification of medically important yeast species by sequence analysis of the internal transcribed spacer regions. J. Clin. Microbiol..

[65] Bougnoux ME, Morand S, D’Enfert C (2002). Usefulness of multilocus sequence typing for characterization of clinical isolates of Candida albicans. J. Clin. Microbiol..

[66] Bougnoux ME (2003). Collaborative consensus for optimized multilocus sequence typing of Candida albicans. J. Clin. Microbiol..

[67] Tavanti A, Gow NA, Senesi S, Maiden MC, Odds FC (2003). Optimization and validation of multilocus sequence typing for Candida albicans. J. Clin. Microbiol..

[68] Odds FC (2007). Molecular phylogenetics of Candida albicans. Eukaryot. Cell..

[69] Tomasini N, Lauthier JJ, Llewellyn MS, Diosque P (2013). MLSTest: novel software for multi-locus sequence data analysis in eukaryotic organisms. Infect. Genet. Evol..

[70] Robles JC, Koreen L, Park S, Perlin DS (2004). Multilocus sequence typing is a reliable alternative method to DNA fingerprinting for discriminating among strains of Candida albicans. J. Clin. Microbiol..

